# Cognitive flexibility's role in shaping self-perception of aging, body appreciation, and self-efficacy among community-dwelling older women

**DOI:** 10.1186/s12912-024-01874-4

**Published:** 2024-04-01

**Authors:** Mona Metwally El-Sayed, Manal Mohammed Hawash, Mahmoud Abdelwahab Khedr, Sarah Ali Hafez, El-Saied Abd El-Hamid Salem, Samir Abdelnaby Essa, Sameer Mohammed Sayyd, Ayman Mohamed El-Ashry

**Affiliations:** 1 Psychiatric and Mental Health Nursing Department, Faculty of Nursing, Alexandria University, Alexandria, Egypt; 2 Gerontological Nursing Department, Faculty of Nursing, Alexandria University, Alexandria, Egypt; 3 Department of Fitness, Gymnastics, and Sports Shows, Faculty of Physical Education for Men, Abu Qir, Alexandria University, Alexandria, Egypt; 4 Department of Physical Education and Sports Sciences, Faculty of Education, Taibah University, Madinah, 41477 Saudi Arabia

**Keywords:** Cognitive flexibility, Self-perception of aging, Body appreciation, Self-efficacy community-dwelling older women, Structural equation modeling

## Abstract

**Background:**

Cognitive flexibility, the capacity to adjust to new information, affects how aging is perceived. In Egyptian culture, older women’s views on aging are shaped by societal gender roles and expectations. These views influence their body image and belief in their abilities, all melded by cultural standards and values.

**Aim:**

Investigate the mediating role of cognitive flexibility in the relationship between self-aging perception, body appreciation, and self-efficacy among community-dwelling older women.

**Methods:**

A correlational analytical design was used on 200 women aged 60 years or older using the Cognitive Flexibility Inventory, Self-Perceptions of Aging, General Self-Efficacy Scale, and Body Appreciation Scales. Structural equation modeling was used in the analysis.

**Results:**

The study found that cognitive flexibility is positively related to self-perception of aging and body appreciation and is also significantly related to general self-efficacy. However, no significant relationship was found between body appreciation and general self-efficacy. Additionally, the study found that cognitive flexibility partially mediates the relationship between self-perception of aging and body appreciation and fully mediates the relationship between body appreciation and self-efficacy.

**Conclusion:**

Cognitive flexibility is vital in the relationships between self-perceptions of aging, body appreciation, and self-efficacy among older women. Therefore, nursing interventions targeting cognitive flexibility are recommended to promote positive self-aging perceptions, body appreciation, and self-efficacy in this population.

## Introduction

Globally, the population is experiencing a significant change in its demographics, marked by a considerable increase in the number of older individuals. The population of 60 and above has been steadily increasing and is expected to rise even more in the coming decades. Egypt is undergoing a demographic shift, with the number of older adults projected to increase from 8.4 million to 22 million between 2020 and 2050. Furthermore, the proportion of women aged 60 and above is also expected to increase [1].

Numerous studies emphasize the importance of comprehending the personal experiences of older women as they age rather than relying solely on objective measures like physical health and social engagement [2, 3]. Self-perception of aging (SPA) refers to an individual's assessment of their aging process, which goes beyond their chronological age [4]. SPA encompasses how people categorize themselves within a specific age group and how old they feel [3]. SPA is a self-fulfilling prophecy, and the age a person feels strongly predicts their well-being and long-term health beyond chronological age [5]. Resilience theory suggests that age-group dissociation, a youthful bias, is a self-protective strategy. According to this theory, negative information about aging amplifies the importance of age in one's self-concept, while positive or neutral information does not have the same effect [6].

In Egyptian society, older women's body image, self-worth, and overall perception of aging can be influenced by traditional gender roles and expectations, beauty standards, marital status, and caregiving roles. SPA can become a self-fulfilling prophecy, and the pressure to maintain youthfulness and beauty can significantly affect older women's body image and how they see themselves as they age. Women often take on caregiving responsibilities within the family, prioritizing the needs of others over their own. This can affect their physical and psychological health and self-perception [7, 8].

Research has shown that older women with positive self-perception and body appreciation tend to have better health outcomes [3] and higher levels of perceived self-efficacy [9]. Furthermore, stereotypes about aging can influence an individual's self-efficacy beliefs and expectations in specific domains [10]. Self-efficacy is a vital personal resource for older women's psychological well-being and physical health, as it allows individuals to approach new tasks and deal with challenging or stressful situations, according to Bandura's theory (2000) [11].

Improving self-efficacy through a positive subjective perception of aging can help older adults deal with age-related challenges [12]. The subjective age of youth is associated with higher memory self-efficacy and increased life satisfaction [13]. A negative SPA is predicted to lead to greater social disconnectedness and depression [14]. Goghari and Lawlor-Savage (2018) found that cognitive training can improve the perception of aging-related changes and cognitive self-efficacy in healthy older adults [15]. Cognitive flexibility, a facet of executive functioning, is pivotal in adapting behavior and problem-solving to evolving aging changes [16]. Cognitive flexibility is crucial for enhancing self-efficacy among older women, which enables them to utilize various strategies to adapt their behaviors and successfully confront challenges in different situations [15, 16].

Nurses can enhance cognitive flexibility among older women, contributing to more positive self-perceptions of aging, body image, and self-efficacy [17]. Addressing ageist stereotypes within Egyptian society, implementing interventions and programs, creating opportunities for social engagement, promoting active and healthy lifestyles [18], and encouraging cognitive training are essential to enhancing well-being among older women [15]. Furthermore, addressing gender-specific issues and societal expectations placed on women concerning aging is crucial [19]. This involves challenging beauty standards, supporting women in maintaining autonomy and independence, and recognizing and valuing the contributions of older women in various domains of life [17]. Creating supportive environments that address the unique needs and experiences of older women can contribute to positive self-perceptions of aging [13].

Despite the importance of cognitive flexibility, there is still a need to understand its mediating role in shaping the perception of older women toward their aging, body image, and self-efficacy. Research in this area has been limited, particularly in the Middle East/North Africa (MENA) region, including Egypt. Therefore, this study aims to investigate the mediating role of cognitive flexibility on the relationship between self-aging perception, body appreciation, and self-efficacy among community-dwelling older women in Egypt, shedding light on the power of mindset in shaping their well-being and overall quality of life.

### Research question

What is the mediating role of cognitive flexibility in the relationship between the self-perception of aging, body appreciation, and self-efficacy of community-dwelling older women?

### Research hypotheses

- ▪ Older women with higher cognitive flexibility are more likely to have a positive self-perception of aging.
- ▪ Older women with higher cognitive flexibility are likelier to have positive body appreciation.
- ▪ Older women with higher cognitive flexibility are more likely to have higher self-efficacy.

## Methods

### Research design

Following the STROBE guidelines, an analytical correlational design was used to investigate the research hypotheses.

### Setting

The El-Wafaa and El-Amal Geriatric Clubs, which cater to older adults in the community and are affiliated with the Ministry of Social Solidarity, were randomly selected from a comprehensive list of geriatric clubs in Alexandria. The study was conducted between October 1 and the end of December 2022. The number of registered older women in the El-Wafaa and El-Amal Geriatric Clubs was 136 and 142, respectively.

### Participants

#### Inclusion and exclusion criteria

The inclusion criteria for this study were as follows: older women had to be 60 years or older, possess coherent communication abilities, and express a willingness to participate. On the other hand, older women who scored 20 on the PHQ-9, indicating severe depressive symptoms, as well as those who scored 0–2 on the Mini-Cog test, suggesting cognitive impairment, were excluded from the study after undergoing screening.

#### Sample size estimation

The participants were estimated using G*Power Windows 3.1.9.7 software with the following parameters: power (1-β err probability) = 0.95, effect size = 0.5, α-error probability = 0.01, number of groups = 1, and predictors = 3. The program determined a sample size of 189 older adult women.

#### Recruitment process and sampling

**Fig. 1:**
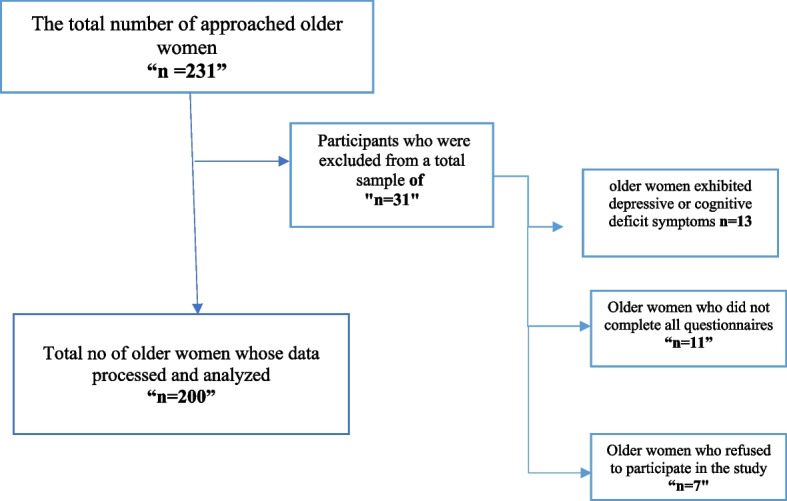
Participants’ recruitment process flow chart

The study was conducted only after obtaining ethical approval from the relevant authorities of the two clubs. A list of registered older women was used for the sampling process generated by software. Using Research Randomizer version 4.0, a simple random sample was selected from this list, which resulted in an initial pool of 231 women. However, 13 participants showing signs of depressive symptoms or cognitive impairments were excluded from the study according to the exclusion criteria. Furthermore, 11 older women only partially completed the interview questions, and 7 others chose not to participate. After applying all the criteria, the final sample consisted of 200 participants who met the study's criteria (Fig. 1).

### Instruments

#### Older women sociodemographic and clinical data sheet

The researchers created a sheet to assess participants' sociodemographic characteristics and clinical profiles. The profile included questions about age, marital status, level of education, employment, place of residence, chronic illnesses, and current medication use.

Two screening instruments were used to exclude older women with severe depressive symptoms or cognitive deficits.

### Patient Health Questionnaire-9 (PHQ-9)

The PHQ-9 is a questionnaire that contains nine items and is self-administered [20]. Each item corresponds to one of the nine DSM-5 depressive symptoms. The questionnaire evaluates how often the client has experienced these symptoms in the previous two weeks, with responses ranging from "0=not at all" to "5=nearly every day." The severity of the symptoms is measured on a scale of 0 to 27, with 5 indicating mild symptoms, 10 indicating moderate symptoms, and 20 indicating severe symptoms. This study used the standardized Arabic version, which had an excellent internal consistency with a Cronbach's alpha score of 0.86 [21].

#### Mini-Cog©

The test assessed cognitive decline among older adults, including a three-item memory recall and a simple clock sketching section [22]. The maximum score is 5, indicating normal cognitive functioning, while a score of 0–2 suggests cognitive impairment and may warrant further evaluation. The standardized Arabic version was adopted in this study, which had a good internal consistency with a Cronbach Alpha of 0.87 [23].

#### Cognitive Flexibility Inventory (CFI)

The CFI is a self-report scale consisting of 20 items to evaluate cognitive flexibility [24]. The scale measures three components of cognitive flexibility: the ability to perceive challenging situations as manageable, recognize various alternative explanations for life events and human behavior, and develop multiple alternative responses to challenging situations. The CFI comprises two subscales: the Alternatives subscale and the Control subscale. Participants are asked to indicate their level of agreement using a 7-point Likert scale ranging from 1 (strongly disagree) to 7 (strongly agree). The total CFI score, as well as the two subscale scores, constitute a score. Higher scores indicate greater cognitive flexibility, with a total score ranging from 20 to 140. Johnco et al. (2014) confirmed the scale's reliability using Cronbach’s alpha values of 0.91 for the Alternatives subscale, 0.86 for the Control subscale, and 0.90 for the total score [25]. The scale was initially translated into Arabic and then backtranslated to ensure accuracy. The content validity of the translated version was assessed using a confirmatory factor analysis, which revealed factor loadings ranging from 0.68 to 0.80 before rotation and from 0.70 to 0.85 after rotation, indicating a strong association between the items and the underlying factors. The CFI demonstrated good internal consistency in our study, with coefficient alpha values of 0.89, 0.91, and 0.88 for the total score, Alternatives, and Control subscales, respectively.

#### Self-Perceptions of Aging (SPA)

The SPA self-rated questionnaire consists of eight questions, with five drawn from the Attitude Towards Own Ageing subscale of the Philadelphia Geriatric Center Morale Scale and three from the Berlin Ageing Study [26, 27]. Respondents rate their level of agreement with each item on a Likert scale ranging from 1 (strongly disagree) to 6 (strongly agree). Positive self-perceptions of aging correspond to higher scores, calculated by reversing negative items and taking the average of all items. Scores on the instrument range from 8 to 48, with scores between 8 and 21 indicating a low level of self-perceptions of aging, scores between 22 and 34 denoting a moderate level, and scores between 35 and 48 representing a higher level. If more than four items have missing values, the final score is set to missing. The study conducted by Sun and Smith in 2017 found high reliability (Cronbach's alpha = 0.81) for the scale [28]. After the SPA questionnaire was translated into Arabic, it underwent evaluation using exploratory factor analysis. Before rotation, the factor loadings ranged from 0.61 to 0.78, but after rotation, they improved and ranged from 0.60 to 0.81, indicating a stronger association between the items and the underlying factors. In the current study, the internal consistency of the total scale was calculated using Cronbach's alpha coefficient test, and the score obtained was 0.82.

#### General Self-Efficacy Scale (GSES)

GSES is a 10-item self-Likert scale to measure a person's overall perception of self-efficacy to estimate how they will handle daily difficulties and adjust to stressful situations [29]. Each item refers to effective coping and suggests an internal, constant attribution of achievement. The Likert-scale rating is on a 4-point scale, where one is not accurate, and four is precisely true (for instance, "Thanks to my resourcefulness, I know how to handle unforeseen situations"). A standard adopted Arabic version was utilized in our study, and it showed internal consistency with a Cronbach's alpha of 0.88 [30]. The scale demonstrated good reliability with α=0.87 in this study.

#### Body Appreciation Scale (BAS-2)

The BAS-2 10-item is a 5-point Likert scale used to measure body image, with responses ranging from 1 (Never) to 5 (Always) [31]. Item responses are summed up to determine a final B.A. score ranging from 5 to 50. Higher scores correspond to greater B.A. In their study, Tylka and Wood-Barcalow (2015) discovered a unidimensional factor structure with intense internal consistency (Cronbach's α = 0.97), construct validity, and test-retest reliability (*r* =0 .90). The study was conducted on a diverse sample of men and women [32]. In our study, the scale was translated into Arabic and back-translated. An exploratory factor analysis was conducted to evaluate its validity. The factor loadings before rotation ranged from 0.67 to 0.77, and after rotation, the factor loadings improved and ranged from 0.68 to 0.83, indicating a stronger association between the items and the underlying factors. The scale demonstrated good internal consistency with a Cronbach's alpha coefficient of 0.89.

### Procedure

#### Ethical approval and consent to participate

The data collection for this study was approved and authorized by the Research Ethical Committee (REC) of Alexandria University. The responsible members of the El-Wafaa and El-Amal Clubs also approved and authorized gathering required information from the selected settings. Participants who wished to participate in the study had to sign a written informed consent form. They were informed that participation was entirely voluntary and that they could withdraw their consent without any negative consequences. The collected information was kept confidential and private.

#### Tool development and validation

The study developed a sociodemographic profile and clinical information for older women. Ineligible older women were excluded using Arabic screening tools (Mini-Cog and PHQ-2). The study measured older women's self-efficacy using the standardized Arabic versions of the GSES. Additionally, the study evaluated the perceptions of aging, body appreciation, and cognitive flexibility using the SPA, BAS-2, and CFI, which were translated into Arabic and back-translated. The study used confirmatory factor analysis to assess the content validity of the translated instruments. The instruments ' content appears valid based on the Lawshe Content Validity Ratio 1. The instruments' accuracy, clarity, relevance, and usability were confirmed. Furthermore, the study's validity was determined by five psychiatric, gerontological, and community health nursing experts who reviewed the instruments.

#### Pilot and reliability

A pilot study was conducted on 20 older women who were not part of the study's participants. The findings of the pilot study indicated that no modifications were required. The Alpha Cronbach's test was utilized to assess the internal consistency of the study's instruments.

#### Data collection

A structured interview schedule was created for each senior woman. The interview was conducted in a clean, private, and comfortable environment to establish trust, clarify the study's objectives, and obtain written consent from each participant. Trained interviewers conducted interviews for approximately 30-35 minutes, designed based on the study's objectives. It included questions about the participants' socio-demographic profiles, clinical information, self-efficacy, perception of aging, body appreciation, and cognitive flexibility.

#### Data analysis and processing

The data was analyzed using IBM SPSS Statistics (version 25) and IBM SPSS AMOS (version 25). Participant demographics were described using frequency and percentage. Quantitative data was summarized using arithmetic mean, standard deviation, Skewness, and Kurtosis. Two-tailed tests were used for all statistical analyses with an alpha error 0.05. A *P*-value of 0.05 or less was considered statistically significant. Path analysis was used to analyze variables. An independent sample t-test and one-way ANOVA analysis of variance were used to identify differences in the study variable based on demographic characteristics. The Mann-Whitney test was used to compare differences between two independent samples when the distributions were not normally distributed, and the sample sizes were small. Kruskal-Wallis's test was used to evaluate the null hypothesis that the 'k' number of samples has been drawn from an identical population with an identical median. Pearson's correlation analysis determined the correlation between significant study variables. Standardized coefficients are used for path analysis of the direct and indirect effects of self-perception of age and body appreciation on general self-efficacy mediated by cognitive flexibility. The Goodness of Fit Index (GFI) ≥ 0.90, and the Adjusted Goodness of Fit Index (AGFI) ≥ 0.90 [33]. The Comparative Fit Index (CFI) is≥ 0.95, and the Tucker-Lewis Index (TLI) is≥ 0.95 [34]. Root Mean Square Error of Approximation (RMSEA) ≤ 0.08 [35].

## Results

Table 1 displays the sociodemographic data of the study subjects. The highest percentage of the participants (37.0%) were between 65 and 68, with an average age of 67.17 (SD= 3.98). More than half of the participants were married (58%) and employed (53%), and 57% reported having a sufficient income. 40% of the subjects had secondary education. Additionally, 26% of the participants lived alone. A significant majority of participants (95%) reported suffering from a chronic illness, with endocrine diseases being the most common (44%), and there was a high usage of endocrine drugs (46.3%).

**Table 1:** Distribution of the study subjects according to their sociodemographic data

**Sociodemographic data**	***n=200***	***%***
**Age**		
61 – 64 years	58	29.0
65 – 68 years	74	37.0
69 – 75 years	68	34.0
**Min. – Max.**	**61-75**	
**M (SD)**	**67.17 (3.98)**	
**Marital Status**		
Married	116	58.0
Single	84	42.0
**Pre-retirement work**		
Housewife	4	2.0
Employee	106	53.0
Entrepreneurial	90	45.0
**Monthly Income**		
Enough	114	57.0
Not enough	86	43.0
**Educational Level**		
Read and write	34	17.0
Primary	36	18.0
Middle school	50	25.0
Secondary	80	40.0
**Place of residence**		
With Partner	116	58.0
With the Children	32	16.0
On my own	52	26.0
**Do you suffer from chronic diseases?**		
Yes	190	95.0
No	10	5.0
**If you suffer from chronic diseases, what are they?**		
Diabetes Meletus	14	7.4
Hypertension	38	20.0
Endocrine diseases	88	46.3
Heart disease	50	26.3
**If you suffer from chronic diseases, do you use any of the following:**		
Hypoglycemic	14	7.4
Antihypertension	34	20.0
Cardiovascular drugs	88	46.3
Endocrine drugs	50	26.3

*M* Mean, *SD* Standard deviation

Table 2 presents the self-perception of age had a mean score of 28.17 (SD= 11.67). The Alternatives subscale had a mean score of 73.17 (SD= 18.44). The Control subscale had a mean score of 32.04 (SD= 13.04). The Cognitive Flexibility Inventory (CFI) had a mean score of 105.21 (SD= 30.82). The General Self-Efficacy Scale (GSES) had a mean score of 28.55 (SD= 9.16). Lastly, the body appreciation had a mean score of 38.41 (SD= 12.02).

**Table 2:** The mean and standard deviation of the self-perception of aging, cognitive flexibility, self-efficacy, and body appreciation among the participants (*n*=200)

**Study Variables**	***Min***	***Max***	***M***	***SD***	***Skewness***		***Kurtosis***	
					***Statistic***	***Std. Error***	***Statistic***	***Std. Error***
**SPA**^**a**^	9	46	28.17	11.67321	-0.318	0.241	-1.251	0.478
**Alternatives Subscale of CF**	34	91	73.17	18.44295	-0.813	0.241	-0.910	0.478
**Control Subscale of CF**	10	48	32.04	13.03912	-0.491	0.241	-1.248	0.478
**CFI**^**b**^	48	139	105.21	30.82479	-0.648	0.241	-1.225	0.478
**GSES**^**c**^	10	40	28.55	9.16336	-0.472	0.241	-0.814	0.478
**BAS-2**^**d**^	16	50	38.41	12.02447	-0.563	0.241	-1.243	0.478

M Mean, SD Standard deviation, *SPA* Self-perceptions of aging, *CFI* Cognitive flexibility inventory, *GSES* General self-efficacy scale, *BAS-2* Body appreciation scale

^a^Higher mean score corresponded to more positive self-perceptions of aging

^b^Higher mean scores indicate more cognitive flexibility

^c^Higher mean scores indicate more self-efficacy

^d^Higher mean scores indicate higher body appreciation

Table 3 illustrates the Pearson correlation coefficients between self-perception of aging, cognitive flexibility, general self-efficacy, and body appreciation. The Alternative and Control subscales and total scores of CF showed a strong positive correlation with self-perception of aging (*r*=0.912, 0.872, and 0.914, *p*<0.01), respectively. General self-efficacy showed strong positive correlations with self-perception of aging (*r*=0.870, *p*<0.01), the Alternatives subscale of CF (*r*=0.917, *p*<0.01), the Control subscale of CF (*r*=0.917, *p*<0.01), and total scores of CF (*r*=0.937, *p*<0.01). Lastly, body appreciation also had strong positive correlations with self-perception of aging, total scores of CF, and general self-efficacy (*r*=0.859, and 0.938 and 0.902, *p*<0.01), respectively.

**Table 3:** Correlation coefficient between self-perception of aging, cognitive flexibility, general self-efficacy, and body appreciation

**Study Variables**		**1**	**2**	**3**	**4**	**5**
**1. Self-perception of aging**	Pearson Correlation					
	Sig. (2-tailed)					
**2. Alternatives Subscale of CF**	Pearson Correlation	0.912^a^				
	Sig. (2-tailed)	0.000				
**3. Control Subscale of CF**	Pearson Correlation	0.872^a^	0.915^a^			
	Sig. (2-tailed)	0.000	0.000			
**4. Total scores of CF**	Pearson Correlation	0.914^a^	0.985^a^	0.970^a^		
	Sig. (2-tailed)	0.000	0.000	0.000		
**5. General Self-Efficacy**	Pearson Correlation	0.870^a^	0.917^a^	0.917^a^	0.937^a^	
	Sig. (2-tailed)	0.000	0.000	0.000	0.000	
**6. Body Appreciation**	Pearson Correlation	0.859^a^	0.922^a^	0.914^a^	0.938^a^	0.902^a^
	Sig. (2-tailed)	0.000	0.000	0.000	0.000	0.000

r: the Pearson correlation coefficient

*CF* Cognitive flexibility

^a^Correlation is significant at the 0.01 level (2-tailed)

Table 4 shows the confirmatory factor analysis (CFA) in the first step to test the structural model. The model fit summary reflected the acceptable level of modeling with X2/df = 1.838, GFI = 0.986, AGFI = 0.892, TLI = 0.989, CFI = 0.998, NFI = 0.995, and RMSEA = 0.092.

**Table 4:** Index evaluation system and relevant structural equation model results

**Model fit**	**X**^**2**^	**df**	**X**^**2**^**/df**	**GFI**	**AGFI**	**TLI**	**CFI**	**IFI**	**RMSEA**
**Value**	3.675	2	1.838	.986	.892	.989	.998	.995	.092
**Acceptable fit**	**-**	-	<3	>0.90	>0.90	>0.95	>0.95	>0.90	<0.08

*X*^*2*^ Chi-Square, *df* Degree of freedom, *GFI* Goodness of fit index, *AGFI* Adjusted goodness of fit index, *TLI* Tucker-lewis index, *CFI* Comparative fit index, *IFI* Incremental fit index, *RMSEA* Root mean square error of approximation

**Fig. 2:**
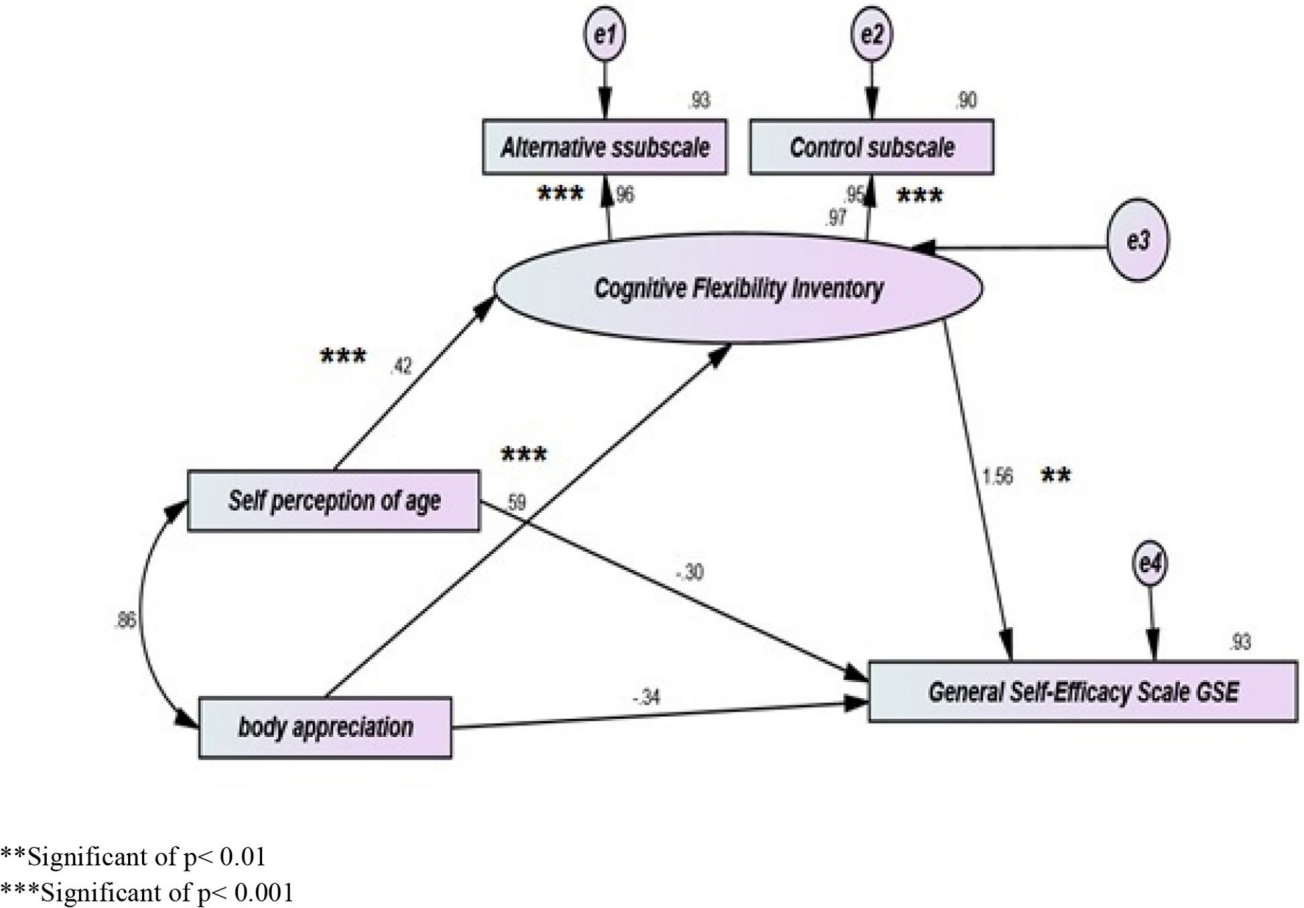
Standardized coefficients for path analysis of the direct and indirect effect of self-perception of age and body appreciation on general self-efficacy mediated by cognitive flexibility

Figure 2 and Table 5 represent the path analysis of the mediation effect of cognitive flexibility on the relationship between self-perception of age, body appreciation, and general self-efficacy. A positive self-perception of aging (β = 0.424) is significantly associated with higher cognitive flexibility (C.R. = 7.663, *p* < 0.001). There is a significant positive relationship between body appreciation (β = 0.594) and cognitive flexibility (C.R. = 10.641, *p* < 0.001). However, the relationship between body appreciation and general self-efficacy is insignificant (C.R. = -1.050, *p* = 0.294). The relationship between self-perception of aging and general self-efficacy is also insignificant (C.R. = -1.275, *p* = 0.202). A significant positive relationship exists between general self-efficacy and cognitive flexibility (C.R. = 2.989, *p* = 0.003). The overall model fit is good, with an X2 of 3.675 and a probability level 0.159. These findings underscore the mediating role of cognitive flexibility in the relationships between self-perception of aging, body appreciation, and general self-efficacy.

**Table 5:** A path analysis of direct and indirect effects of self-perception of aging and body appreciation on general self-efficacy mediated by cognitive flexibility

			**Standardized Regression Weight**	**Estimate**	**S.E.**	**C.R.**	**P**
**Cognitive Flexibility**		Self-perception of aging	.424	.647	.084	7.663	***
**Cognitive Flexibility**		Body Appreciation	.594	.879	.083	10.641	***
**General Self Efficacy**		Body Appreciation	-.337	-.257	.245	-1.050	.294
**General Self Efficacy**		Self-Perception of aging	-.302	-.237	.186	-1.275	.202
**Alternatives Subscale of CF**		Cognitive Flexibility	.964	1.000			
**Control Subscale of CF**		Cognitive Flexibility	.949	.696	.030	22.865	***
**General Self Efficacy**		Cognitive Flexibility	1.563	.805	.269	2.989	.003

Model X2=3.675

Probability level = .159

*S.E.* Standard error, *C.R*. Critical ratio

^***^Significant of *p*< 0.001

## Discussion

The interplay between self-perception of aging, body appreciation, and self-efficacy significantly shapes older women's well-being and quality of life. Cognitive flexibility, the ability to adapt to new information and changing situations, is pivotal in this interplay between self-perception of aging, body appreciation, and self-efficacy. Older women must adapt quickly to changing situations due to new information and environmental demands [32, 36].

This study found that the older women studied exhibited high cognitive flexibility. Consistent with these results, it has been proposed that individuals possess varying 'brain ages,' leading to differences in functional brain maturity and higher cognitive flexibility among age-matched individuals [37]. Furthermore, it was found that older women outperform men in cognitive flexibility in verbal tasks such as verbal fluency and memory [38]. Conversely, several research studies have reported that older adults often exhibit less cognitive flexibility, commonly attributed to an age-related decline in acquiring and updating information [39, 40] and age-related changes in brain dynamics [41].

Aging typically manifests in various physical and cognitive changes and challenges that are not always readily accepted [42]. The current results revealed moderate self-aging perception, body appreciation, and self-efficacy among the studied older women, which is considered an optimistic finding. This could be interpreted by the fact that older adults today are healthier, have better functioning, and are more active than their counterparts in previous years, which is reflected in these areas. Supporting the current results, Barbaccia et al. (2022) found that mature and older adults perceive the concept of active aging positively [43]. Their qualitative study found that older adults want to re-engage in life, continue to be active, and maintain their social life and independence, indicating an appropriate level of self-efficacy. On the contrary, further research studies found that the prevalent functional decline, frailty, and disability associated with aging negatively affect self-aging perception, body appreciation, and self-efficacy in later life [12, 44].

The current pathway analysis aimed to investigate the mediating role of cognitive flexibility in the relationship between self-aging perception, body appreciation, and self-efficacy among community-dwelling older women. The regression analysis revealed that self-perception of age and body appreciation were significant predictors of cognitive flexibility in these women. Furthermore, cognitive flexibility mediated the relationship between self-perception of age, body appreciation, and self-efficacy.

Firstly, the positive and significant correlation between older women's self-perception of age and cognitive flexibility could be attributed to individuals developing cognitive flexibility during aging by reflecting, interpreting, perceiving, and attributing meaning to experienced age changes [45]. Secondly, the positive and significant correlation between older women's body appreciation and cognitive flexibility could be due to the cognitive process affecting how individuals perceive themselves, including their body image and appreciation. In line with the current findings, it has been reported that body dissatisfaction and unfavorable social comparisons are significant risk factors for psychological distress and cognitive inflexibility [46]. It has also been documented that interventions focusing on cognitive inflexibility could help treat symptoms associated with body shape dissatisfaction, thereby reducing negative body image [47]. Moreover, a study measuring the physical fitness and cognitive functions of 72 older individuals indicated that agility, body appreciation, and body balance were significantly associated with cognitive flexibility in older adults [48].

Finally, cognitive flexibility was a crucial mediator in the relationship between self-aging perception, body appreciation, and self-efficacy. This underscores the importance of cognitive adjustment and flexibility in balancing older women's views and perceptions of themselves and adapting to age-related changes. This will enhance older women's self-efficacy and their beliefs in their ability to perform the actions needed to achieve specific goals. Similarly, major lifespan theories related to old-age challenges support this proposed explanation regarding the psychological path from SPA to self-efficacy and physical functioning [45]. Also, Lee (2011) reported that self-efficacy remained significantly correlated with physical condition, attractive body, and cognitive flexibility among older adults [49]. Furthermore, other studies found that individuals with more positive self-perceptions of aging tend to maintain appropriate levels of self-efficacy over time [11, 12].

### Limitations of the study

Despite the excellent fit of the model, it is essential to acknowledge the study's potential limitations. One such limitation is that the study only included a specific group of community-dwelling older women, which could affect the generalizability of the findings to other populations. However, this selection helped minimize the confounding variables' impact and reinforce the self-perception of aging among older women. Additionally, the use of self-reported measures in the study may not have fully captured the participants' actual experiences and could be subject to bias.

### Conclusion and recommendations

Based on our findings, cognitive flexibility is crucial in how older women perceive themselves and adapt to age-related changes. The results suggest that maintaining a positive perception of one’s aging process and appreciating one’s body can lead to higher levels of self-efficacy, thereby improving physical functioning. Moreover, positive self-perceptions of aging are associated with sustained high levels of self-efficacy over time. These findings underscore the importance of cognitive adjustment and flexibility in promoting healthy aging practices and fostering positive self-perceptions among older adults.

### Nursing implications

The findings of this study have several implications for nursing practice. Nurses could develop training programs such as Cognitive Behavioral Therapy (CBT), Mindfulness-based Practices, and Acceptance and Commitment Therapy (ACT) to enhance cognitive flexibility in older women, helping them adapt to age-related changes and maintain a positive self-perception. Understanding the role of cognitive flexibility in self-perception and physical functioning can help nurses provide holistic care that addresses both physical and psychological aspects of aging. Additionally, nurses can educate older women about the importance of positive self-perception and body appreciation in maintaining high levels of self-efficacy. These strategies highlight the importance of cognitive adjustment and flexibility in promoting healthy aging practices and positive self-perceptions among older women.

## Data Availability

The datasets used and analyzed during the current study are available from the corresponding author upon reasonable request.

## References

[1] Organization WH. Aging and health (2021). https://www.who.int/newsroom/factsheets/detail/ageing-and-health. It was accessed 29 Jan 2022.

[2] Tully-Wilson C, Bojack R, Millear PM, Stallman HM, Allen A, Mason J (2021). Self-perceptions of aging: a systematic review of longitudinal studies. Psychol Aging.

[3] Blawert A, Krumpoch S, Freiberger E, Wurm S (2021). Domain-specific self-perceptions of aging are associated with different gait patterns in older adults: a cross-sectional latent profile analysis. BMC Geriatric.

[4] Hughes ML, Touran DR (2021). Aging in context: Incorporating everyday experiences into the study of subjective age. Front Psychiatry.

[5] Tully-Wilson C, Bojack R, Millear PM, Stallman HM, Allen A, Mason J (2021). Self-perceptions of aging: a systematic review of longitudinal studies. Psychol Aging.

[6] Pinquart M (2002). Good news about the effects of bad old-age stereotypes. Exp Aging Res.

[7] Sabatini S, Siebert JS, Diehl M, Brothers A, Wahl HW (2022). Identifying predictors of self-perceptions of aging based on a range of cognitive, physical, and mental health indicators: twenty-year longitudinal findings from the ILSE study. Psychol Aging.

[8] Ahmed D, El Shair IH, Taher E, Zyada F (2014). Prevalence and predictors of depression and anxiety among the elderly population living in geriatric homes in Cairo, Egypt. J Egypt Public Health Assoc.

[9] Bandura A, Freeman WH, Lightsey R (1997). Self-efficacy: the exercise of control. J Cogn Psychother.

[10] Karns BM. Effect of age-related stereotypes on self-efficacy and self-perception. Texas Woman’s University; 2003. https://digitalcommons.wku.edu/theses/670.

[11] Bandura A (2000). Self-efficacy: the foundation of agency. Control of human behavior, mental processes, and consciousness: essays in honor of the 60^th^ birthday of August Flammer.

[12] Tovel H, Carmel S, Raveis VH (2019). Relationships among self-perception of aging, physical functioning, and self-efficacy in late life. J Gerontol Series B.

[13] Stephan Y, Caudroit J, Chalabaev A (2011). Subjective health and memory self-efficacy as mediators in the relation between subjective age and life satisfaction among older adults. Aging Ment Health.

[14] Bryant C, Bei B, Gilson K, Komiti A, Jackson H, Judd F (2012). The relationship between attitudes to aging and physical and mental health in older adults. Int Psychogeriatric.

[15] Goghari VM, Lawlor-Savage L (2018). Self-perceived benefits of cognitive training in healthy older adults. Front Aging Neurosci.

[16] Boron JB, Haavisto W, Willis S, Robinson P, Schaie K (2018). Longitudinal change in cognitive flexibility: impact of age, hypertension, and APOE4. Innov Aging.

[17] Benyamini Y, Burns E (2020). Views on aging: older adults’ self-perceptions of age and of health. Eur J Ageing.

[18] McAuley E, Konopack JF, Morris KS, Motl RW, Hu L, Doerksen SE (2006). Physical activity and functional limitations in older women: influence of self-efficacy. J Gerontol B Psychol Sci Soc Sci.

[19] Luo MS, Li LW, Hu RX (2021). Self-perceptions of aging and domain-specific health outcomes among midlife and later-life couples. J Aging Health.

[20] Spitzer RL, Kroenke K, Williams JB (1999). Validation and utility of a self-report version of PRIME-MD: the PHQ primary care study. Primary care evaluation of mental disorders. Patient Health Questionnaire. JAMA.

[21] AlHadi AN, AlAteeq DA, Al-Sharif E, Bawazeer HM, Alanazi H, Al-Shomrani AT (2017). An Arabic translation, reliability, and validation of Patient Health Questionnaire in a Saudi sample. Ann Gen Psychiatry.

[22] Borson S, Scanlan J, Brush M, Vitaliano P, Dokmak A (2000). The mini cog: a cognitive ‘vital signs’ measure for dementia screening in multi-lingual elderly. Int J Geriatr Psychiatry.

[23] Albanna M, Yehya A, Khairi A, Dafeeah E, Elhadi A, Rezgui L (2017). Validation and cultural adaptation of the Arabic versions of the Mini-Mental Status Examination–2 and Mini-cog test. Neuropsychiatr Dis Treat.

[24] Dennis JP, Vander Wal JS (2010). The cognitive flexibility inventory: Instrument development and estimates of reliability and validity. Cognit Ther Res.

[25] Johnco C, Wuthrich VM, Rapee RM (2014). Reliability and validity of two self-report measures of cognitive flexibility. Psychol Assess.

[26] Lawton MP (1975). The Philadelphia geriatric center morale scale: a revision. J Gerontol.

[27] Liang J, Bollen KA (1983). The structure of the Philadelphia Geriatric Center Morale scale: a reinterpretation. J Gerontol.

[28] Sun JK, Smith J (2017). Self-perceptions of aging and perceived barriers to care: Reasons for health care delay. Gerontologist.

[29] Schwarzer R, Jerusalem M. Generalized self-efficacy scale. J Weinman, S Wright, M Johnston. Measures in health psychology: a user’s portfolio Causal and control beliefs. 1995;35:37. 10.1037/t00393-000.

[30] Crandall A, Rahim HA, Yount KM (2015). Validation of the general self-efficacy scale among Qatari young women. East Mediterranean Health J.

[31] Tylka TL, Wood-Barcalow NL (2015). The Body Appreciation Scale-2: item refinement and psychometric evaluation. Body Image.

[32] Richard’s MM, Marino JC. Flexibilidad cognitiva, una capacidad esencial: ¿ cambio cognitivo, propiedad dinámica o" pago de costes por alternancia? 2016. https://ri.conicet.gov.ar/handle/11336/110207.

[33] Kline RB (2015). Principles and practice of structural equation modeling.

[34] Hu LT, Bentler PM (1999). Cutoff criteria for fit indexes in covariance structure analysis: conventional criteria versus new alternatives. Struct Equa Model.

[35] Browne MW, Cudeck R, Bollen KA, Long JS (1993). Alternative ways of assessing model fit. Testing structural equation models.

[36] Richard’s MM, Krzemien D, Valentina V, Vernucci S, Zamora EV, Comesaña A (2021). Cognitive flexibility in adulthood and advanced age: Evidence of internal and external validity. Appl Neuropsychol Adult.

[37] Dosenbach NUF, Nardos B, Cohen AL, Fair DA, Power JD, Church JA (2010). Prediction of individual brain maturity using fMRI. Science.

[38] Heinzel S, Metzger FG, Ehlis AC, Korell R, Alboji A, Haeussinger FB (2013). Aging-related cortical reorganization of verbal fluency processing: a functional near-infrared spectroscopy study. Neurobiol Aging.

[39] Wilson CG, Nusbaum AT, Whitney P, Hinson JM (2018). Age differences in cognitive flexibility when overcoming a preexisting bias through feedback. J Clin Exp Neuropsychol.

[40] Di X, Gohel S, Thielcke A, Wehrl HF, Biswal BB, Initiative ADN (2017). Do all roads lead to Rome? A comparison of brain networks derived from inter-subject volumetric and metabolic covariance and moment-to-moment hemodynamic correlations in old individuals. Brain Struct Funct.

[41] Kupis L, Goodman ZT, Kornfeld S, Hoang S, Romero C, Dirks B (2021). Brain dynamics underlying cognitive flexibility across the lifespan. Cerebral Cortex.

[42] Sánchez-Cabrero R, León-Mejía AC, Arigita-García A, Maganto-Mateo C (2019). Improvement of body satisfaction in older people: An experimental study. Front Psychol.

[43] Barbaccia V, Bravi L, Murmura F, Savelli E, Viganò E (2022). Mature and older adults’ perception of active ageing and the need for supporting services: Insights from a qualitative study. Int J Environ Res Public Health.

[44] Börsch-Supan A, Kneip T, Litwin H, Myck M, Weber G, editors. Ageing in Europe: Supporting policies for an inclusive society. Berlin: de Gruyter; 2015.

[45] Wurm S, Westerhof GJ (2015). Longitudinal research on subjective aging, health, and longevity: current evidence and new directions for research. Annu Rev Gerontol Geriatr.

[46] Rodgers RF, DuBois RH (2016). Cognitive biases to appearance-related stimuli in body dissatisfaction: a systematic review. Clin Psychol Rev.

[47] Selvi K, Parling T, Ljótsson B, Welch E, Ghaderi A (2021). Two randomized controlled trials of the efficacy of acceptance and commitment therapy-based educational courses for body shape dissatisfaction. Scand J Psychol.

[48] Christensen JR, Faber A, Ekner D, Overgaard K, Holtermann A, Søgaard K (2011). Diet, physical exercise and cognitive behavioral training as a combined workplace-based intervention to reduce body weight and increase physical capacity in health care workers a randomized controlled trial. BMC Public Health.

[49] Lee C. Self-efficacy and physical activity in older adults. Ball State University. 2011. 10.1177/2F1559827610392704.

